# Soft Microwave Pretreatment to Extract *P*-Hydroxycinnamic Acids from Grass Stalks

**DOI:** 10.3390/molecules24213885

**Published:** 2019-10-28

**Authors:** Aurélie Bichot, Mickaël Lerosty, Laureline Geirnaert, Valérie Méchin, Hélène Carrère, Nicolas Bernet, Jean-Philippe Delgenès, Diana García-Bernet

**Affiliations:** 1 Univ Montpellier, INRA, 102 Avenue des Etangs, CEDEX, 11100 Narbonne, France; 2 INRA Institut Jean-Pierre Bourgin, CEDEX, 78026 Versailles, France

**Keywords:** microwave pretreatment, grass biomass, *p*-hydroxycinnamic acids extraction

## Abstract

The aim of this article is to provide an analysis of microwave effects on ferulic and coumaric acids (FA and CA, respectively) extraction from grass biomass (corn stalks and miscanthus). Microwave pretreatment using various solvents was first compared to conventional heating on corn stalks. Then, microwave operational conditions were extended in terms of incident power and treatment duration. Optimal conditions were chosen to increase *p*-hydroxycinnamic acids release. Finally, these optimal conditions determined on corn stalks were tested on miscanthus stalks to underlie the substrate incidence on *p*-hydroxycinnamic acids release yields. The optimal conditions—a treatment duration of 405 s under 1000 W—allowed extracting 1.38% FA and 1.97% CA in corn stalks and 0.58% FA and 3.89% CA in miscanthus stalks. The different bioaccessibility of these two molecules can explain the higher or lower yields between corn and miscanthus stalks.

## 1. Introduction

Lignocellulosic biomass is a key resource for the sustainable development of the bioeconomy, and its effective implementation would allow a decisive matter and energy co-valorization. First, it is available on all continents in different forms and species. Second, the volumes of available resources are very large, represent an interesting carbon source, and come from numerous sustainable sources avoiding land-use conflicts: agricultural and agri-food wastes, wastes from land-use planning, forest woody residues, perennial crops on polluted soils (miscanthus), algae, or municipal solid wastes [1]. Nevertheless, the use of lignocellulosic biomass is curtailed because of high processing costs and difficulties related to obtaining high extraction yields. Nowadays, many applications (food and chemical industry, materials) are being explored from agricultural residues and miscanthus, as they represent an alternative source of many high-value molecules (sugars, ethanol, acetic acid, butyric acid, xylitol, propionic acid, oil, etc.) and fibers [2].

**Figure 1:**
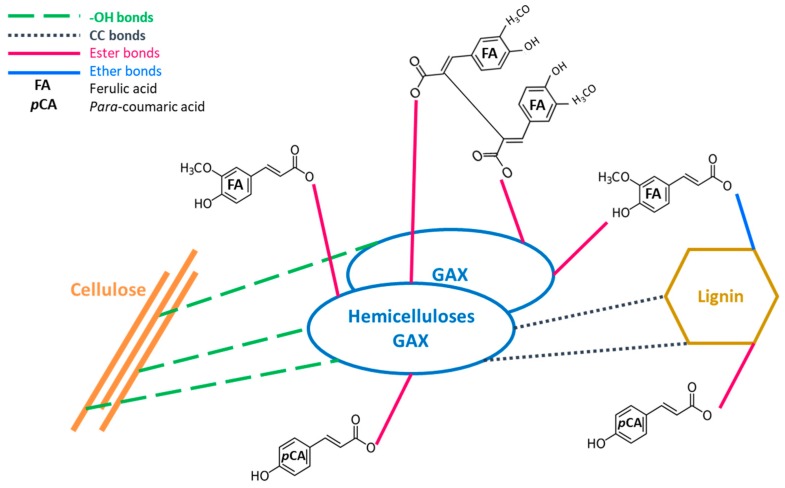
Global organization of grass cell wall.

Among them, *p*-hydroxycinnamic acids are increasingly attracting the attention of scientists and industry because of their interesting features. Indeed, due to their anti-oxidant properties, *p*-hydroxycinnamic compounds can be used as food preservatives and have a preventive role in some types of cancer [3]. Ferulic, coumaric, syringic, and caffeic acids are the most commonly found acids in cereals, whether in grains or stems [4]. They are covalently bounded to cell walls and difficult to access. Indeed, *p*-coumaric acid is principally esterified on lignin S units, and ferulic acid is both etherified to lignin and esterified to hemicelluloses [5,6,7]. Figure 1 graphically represents the different chemical bonds that form the complex three-dimensional biomass network.

Many factors can explain *p*-hydroxycinnamic acids’ accessibility and lignocellulosic biomass resistance to degradation: polymers composition and/or organization, protein and acetyl group abundance, specific surface area, or a combination of all these factors [8]. Three main polymers—cellulose, hemicelluloses, and lignin—compose lignocellulosic biomass [9]. While lignin is believed to play an important role in cellulose protection from hydrolysis to sugar monomers by forming a physical barrier and adsorbing enzymes [10], other key factors such as hemicelluloses rate, cellulose crystallinity, acetylation, or porosity are supposed to hinder cellulose hydrolysis by forming a complex and solid lignocellulose network [11]. Thus, applying an efficient pretreatment to break down lignocellulosic biomass in order to favor bioconversions and facilitate access to molecules of interest is mandatory to obtain an economically sustainable process.

Many pretreatments have been tested in order to reduce lignocellulose recalcitrance to *p*-hydroxycinnamic acids release, including: biological, chemical, or physical treatment [12,13]. The present article is focusing on microwave pretreatment, which is a thermal treatment that has gained interest over the past 10 years due to its numerous advantages. Indeed, microwave treatment heats material directly without any contact, shortens reaction time by reaching high temperatures faster than conventional heating systems, increases yields and purity by reducing the formation of side products and inhibitors, diffuses homogenous microwave irradiation inside the cavity, thus allowing high replication, consumes less solvent, and allows a quick control of the operating parameters [14,15]. Due to these potential benefits, many recent studies are devoted to the application of this technology to improve *p*-hydroxycinnamic acids and sugars release from lignocellulosic biomass and/or waste activated sludge biodegradability [16]. A systemic approach is proposed: after testing large range of operating parameters (solvent, duration, power, power density) on a specific biomass (corn stalks), the conditions that lead to the highest *p*-hydroxycinnamic acids yields were extended on another lignocellulosic biomass.

## 2. Results and Discussion

### 2.1. Raw Matter Composition

Before performing any treatment, dry matter content (DM) was estimated according to the NREL (National Renewable Energy Laboratory from US Department of Energy) protocol. Table 1 presents parietal composition of raw materials expressed in % DM. Parietal composition corresponds to all the elements that are part of the biomass cell wall: hemicellulose, cellulose, lignin, and ash. Van Soest protocol was used to determine hemicelluloses, cellulose, acid detergent lignin (ADL), and ash in a gravimetrical way, and NREL protocol was used to determine Klason lignin. A clear difference can be seen in the composition of the two studied biomass samples, particularly in terms of wall proportion. Soluble content in miscanthus stalks is very low, and 95% DM of the mass consists of the cell wall, of which cellulose and lignin represent a significant part. Miscanthus is lignified to a greater extent than corn stalks (twice as much), and its cellulose content is two times higher. It appears that the compositions of the two biomasses differ significantly, which could explain the differences in behavior following microwave treatment.

Biomass composition (*p*-hydroxycinnamic acids, parietal polymers) was in agreement with values found in the literature. Provan et al. [17] worked on phenolic compounds in maize straw (2 g CA/100 g DM, 0.6 g FA/100 g DM) or wheat straw (0.5 g CA/100 g DM, 0.01 g FA/100 g DM). In terms of parietal polymers, the composition can vary significantly among species, but generally admitted values vary between 27%–40% DM cellulose, 25%–34% DM hemicellulose, and 9%–15% DM lignin for corn stalks, and 28%–49% DM cellulose, 24%–32% DM hemicellulose, and 15%–28% DM lignin for miscanthus stalks [11].

### 2.2. Effect of Solvent and Treatment on p-Hydroxycinnamic Acids Release

Preliminary experiments were performed on corn stalks sample F98902 using one microwave condition (500 W for 270 s) and different solvents, in order to select the best one for the pretreatment of biomass with the microwave pilot. Indeed, solvent is one of the most important parameters in microwave pretreatments, as it affects the solubility of the target components, and it interacts differently with microwaves depending on its polarity. Polar solvents absorb microwaves, allowing reaction media to reach high temperatures quickly, and provoking cell disruption, thus facilitating the release of molecules of interest; while non-polar solvents are transparent to microwaves [15,18,19]. In the literature, many solvents have been tested, including non-polar solvents that do not absorb waves and allow waves to be concentrated on material. To our knowledge, no scientists have worked with non-polar solvents in the microwave pretreatment of biomass studies, but hexane is commonly used mixed with a polar solvent, such as water, for the treatment of polluted soils [20]. Concerning polar solvents, water and ethanol mixtures are the most commonly used. With ethanol 50% (*w:w*) and 30 W, Carniel et al. [18] extracted 3.74 mg gallic acid equivalent/g *Physalis angulate* in less than a minute. Oufnac et al. [21] extracted with methanol up to 467 µg catechin equivalent/g wheat bran, which is twice as high as the extraction yield obtained using conventional heating.

In this study, only polar solvents were tested, including water, alkaline water (NaOH), acidic water (H_2_SO_4_), and water/ethanol mixture. The operating conditions were the following (Table 2): 270 s of microwave treatment under 500 W (0.179 Wh/g), 360 s of conventional heating, or control without any heating. pH was measured after one hour of soaking, just before treatment. No significant change in pH was observed after the treatment (Table 2). The antioxidant activities were not studied in this study and considered equivalent between samples, meaning that the biological activity was assessed as not having a significant impact on the biomass during the treatments. The maximum temperature was 99 °C for microwave pretreatments in water, 80 °C for microwave pretreatment in ethanol, and between 66 °C and 75 °C for conventional pretreatments, depending on solvents. Therefore, conventional processing involved a much longer heating time, which is consistent with the bibliographic references: Del Rio et al. [22] realized a biomass thermal treatment for 20 min to reach 120 °C.

#### 2.2.1. Composition Analysis of Pretreated Biomass

**Figure 2:**
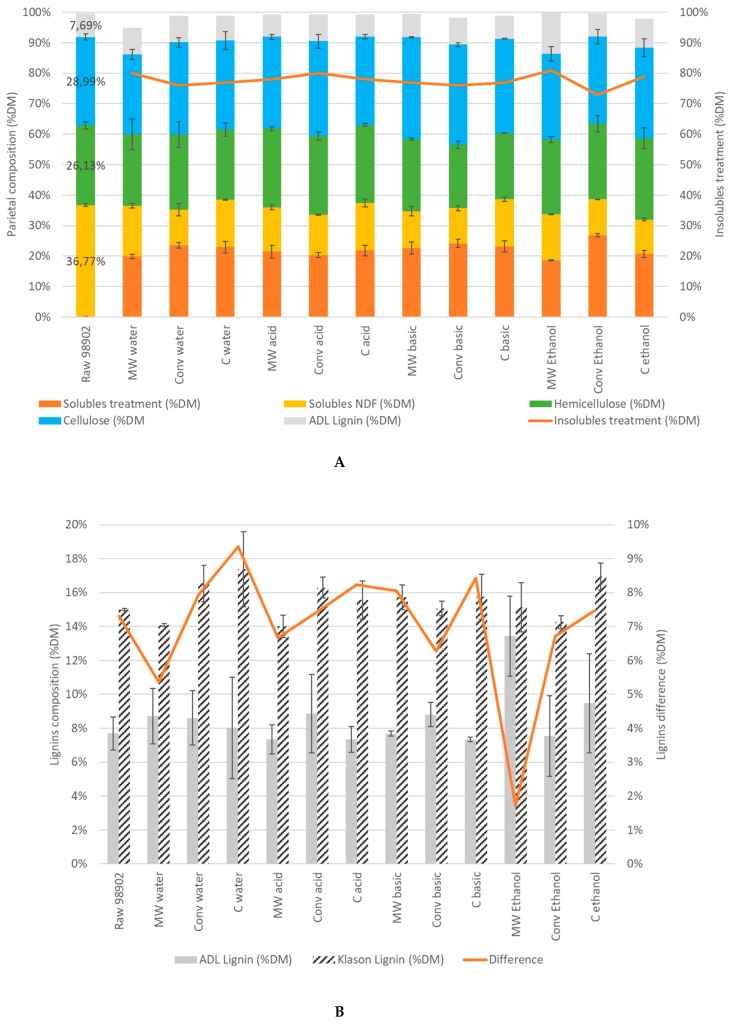
Biomass parietal composition depending on treatment (%DM) with Van Soest method (**A**). The figures in bold (**A**) are the percentages of insoluble content following the treatments (the mass of solid recovered after drying at 40 °C); the ash content is not represented. Comparison between Klason lignin and ADL (**B**). The figures in bold (**B**) are the differences between the two lignins measurements. Means of triplicates.

Biomass composition was characterized using Van Soest and Klason methods (Figure 2). According to Figure 2A, parietal polymers contents are not statistically different between raw matter and pretreated biomass: standard deviations ranging from 0.1% to 2.3% intersect and do not lead to significant differences in parietal composition due to treatments (ANOVA results not shown). For all treatments tested, ADL represents 7.6% DM, cellulose represents 29% DM, hemicellulose represents 26% DM, and total soluble content (from treatment and from NDF Van Soest first step) represents 36.7% DM. Corn stalks’ parietal composition is not significantly modified by the treatments tested.

The lignin content estimated by two different analytical methods is presented in Figure 2B. From statistical analysis, the treatment or the solvent do not statistically affect lignins (ADL and Klason). Lignin is a very resistant polymer to be impacted by the tested soft microwaves conditions. By looking in greater detail, Klason lignin content is almost always twice as high as ADL content [23], and the differences between the two calculation methods vary between 5.4% and 9.4% (excepted for ethanol microwave treatment, for which the difference is much smaller with 1.7%). As explained earlier, this difference could be interpreted as the part of the lignin linked by β-O-4 bonds and solubilized in 72% H_2_SO_4_. As this difference does not vary with the type of treatment employed, it means that β-O-4 bonds are not affected by the treatments, and it can be assumed that the lignin internal structure remains the same.

The results differ from those obtained by Choudhary et al. [24]. When sorghum was pretreated with alkaline water up to 0.2 g NaOH/g DM in 20 mL water for 4 min and 1000 W, 58.9% lignin was removed, and 54.9% of hemicellulose was removed, but the cellulose content never varied. In our study, the minor changes in biomass structure can be explained by the moderate temperature reached, which never exceeded 100 °C, by the low incident power during the tests (500 W). Moreover, in Choudhary et al. [24], the NaOH concentration was high compared to our study, and explains lignin degradation and hemicellulose solubilization.

The total mass recovered after treatments of 10 g was around 8 g (Figure 2A). Acid, alkaline, or ethanol did not modify the solubility of components: in all cases tested, 2 g of raw matter (out of 10 g used) were solubilized during the treatment.

#### 2.2.2. *p*-Hydroxycinnamic Acids Release

According to the literature, an increased extraction of *p*-hydroxycinnamic acids in the presence of NaOH was expected due to the ester linkages first between ferulic acid and hemicelluloses and second between *p*-coumaric acid and S lignin units, which correspond to the cleavage of the ester bonds and α-aryl ether bonds rupture linking *p*-hydroxycinnamic acids to the cell wall [25]. In contrast, acid treatment should break the glycosidic bonds and favor sugar solubilization while leaving the ester bonds intact [26]. An analysis of the *p*-hydroxycinnamic acids released in the liquid phase during the different pretreatments performed is summarized in Table 2.

From Table 2, it appears that the FA and CA recovered in the liquid fraction after treatments varied slightly with the experimental conditions tested in this study. The highest CA extraction values were observed with ethanol and alkaline water as solvent and microwave heating as treatment (0.298 mg CA/g DM and 0.247 mg CA/g DM, respectively). In case of FA, alkaline water appeared to be the most effective solvent without the microwave heating further increasing efficiency.

In the chosen conditions, alkaline treatment ensured the highest yields compared to the other solvents tested. Moreover, the NaOH concentration was similar to the one employed by Mussatto et al. [27]. Indeed, Mussatto et al. [27] carried out a conventional treatment of acid pretreated brewer’s spent grain. They extracted up to 4.27 mg FA/g DM and 4.08 mg CA/g DM after 90 s at 120 °C using 2% NaOH (*w/v*). In that case, no microwave treatment was investigated, but the temperature and the previous acid pretreatment were the main parameters affecting FA and CA release.

The total initial amounts of FA and CA in raw corn stalks were 4 mg/g DM and 13 mg/g DM, respectively. Therefore, *p*-hydroxycinnamic acids release yields did not exceed 2.3% with all the tested solvents in the studied conditions (500 W, 270 sec). Moreira et al. [28] found approximately the same extraction yields from brewer’s spent grain: one gram treated with 20 mL of NaOH 0.75% for 15 min and 100 °C in a microwave oven permitted approaching a yield of 1.31% of FA. These relatively low yields are compared to the high ones obtained with 2 mL of NaOH 2N protocol on 20 mg of dry matter for a night: in this latter case, the percentage of NaOH is much higher and reaches 88% g/g DM. Even if no heating is applied during the 24 h of reaction, the reagent concentration is so high that the ester bonds are largely degraded. In the present study, such high NaOH concentrations are not desired in order to develop an environmentally friendly process.

Moreover, in our study, according to ANOVA (Table 3), the solvent had more impact on FA release than the treatment itself (p = 1.13 × 10^−7^ and p = 0.0704 for solvent and treatment effects respectively). On the contrary, the release of CA was significantly dependent on solvent (p = 5.07 × 10^−7^) and on treatment (p = 2.7 × 10^−7^). Finally, the interaction between the solvent and treatment did not have a significant effect on *p*-hydroxycinnamic acids release (p > 0.05%).

Another ANOVA was performed to determine the effect of each type of treatment and solvents: conventional heating did not significantly favored FA extraction (p = 0.286) compared to the control. Microwave treatment had a significant negative effect on FA release with t value = −2.479. This could be explained by a negative effect of waves on biomass structure and *p*-hydroxycinnamic acids organization. Acid solvent as well was significantly disadvantageous for the extraction of FA (t value = −4.394) compared to water alone. When water was used as solvent during microwave treatment, Diaz et al. [29] demonstrated that neither lignin nor cellulose were modified, and that only a small amount of hemicellulose content was reduce; this may explain the negative effects on *p*-hydroxycinnamic acid release in our case with water. A similar or lower yield is naturally expected with acid as solvent.

From Table 2, for both FA and CA, the alkaline solvent allows an increase in acids release compared to other solvents for the same treatment, because a break-up of ester bonds linking *p*-hydroxycinnamic acids to the cell wall is favored with alkali [26].

Concerning *p*-hydroxycinnamic acids release, microwave pretreatment efficiency is variable according to the literature: recovered molecules yields vary a lot depending on the solvent and raw matter. In this way, Carniel et al. [18] demonstrated that to release a maximum amount of total phenolic compounds (TPC), the most important parameter is the solvent and using ethanol 50% in volume allowed to obtain 3.74 mg TPC/g DM after extraction from *Physalis angulate* seeds. Concerning maize, this substrate is often cited in the literature, but usually the microwave is used to degrade the cell wall and gain access to polysaccharides, and only the evolution of parietal polymers composition is analyzed. Wheat bran was treated by microwave (500 W, 20 min 100 °C) with methanol by Oufnac et al. [21], but even if the incident power and final temperature were the same as in this study, the results are difficult to compare: treatment duration was more than four times longer, and efficiency is measured by catechin equivalent release (467 µg catechin equivalent/g wheat bran). Catechin is part of the flavonoids molecules family and is responsible for the color of the plant. Since this molecule is not linked to cell walls and is hydrolysable, it reacts to microwaves differently than *p*-hydroxycinnamic acids that are covalently linked to cell walls.

In order to better understand microwave effects on *p*-hydroxycinnamic acids release, it was decided to focus on water, which is considered as a suitable extraction solvent: Moreira et al. [28] found it to be the second best solvent to extract polyphenols behind NaOH at 150 °C for 25 min. Moodley and Kana [30] demonstrated that NaOH concentration has more impact than microwave duration on sugars release. Thus, by working with water, NaOH effects are constrained, which allows better understanding the thermal and non-thermal microwave effects. Finally, using water as solvent is environmentally friendly and appropriate from an industrial application point of view: water is low cost compared to other solvents, and wastewater management is much easier without chemical reagent addition.

Therefore, the optimization of microwave treatment on corn stalks with water as solvent was performed using a response surface methodology (RSM). The best conditions were then applied to miscanthus.

### 2.3. Response Surface Methodology (RSM) Analysis to Determine Better Microwave Parameters

RSM analysis was used to determine the optimal operating conditions in terms of FA and CA release from corn stalks. Moreover, the lignocellulosic biomass structure was also analyzed through the Van Soest and Klason lignin methods.

#### 2.3.1. Experimental Design

**Figure 3:**
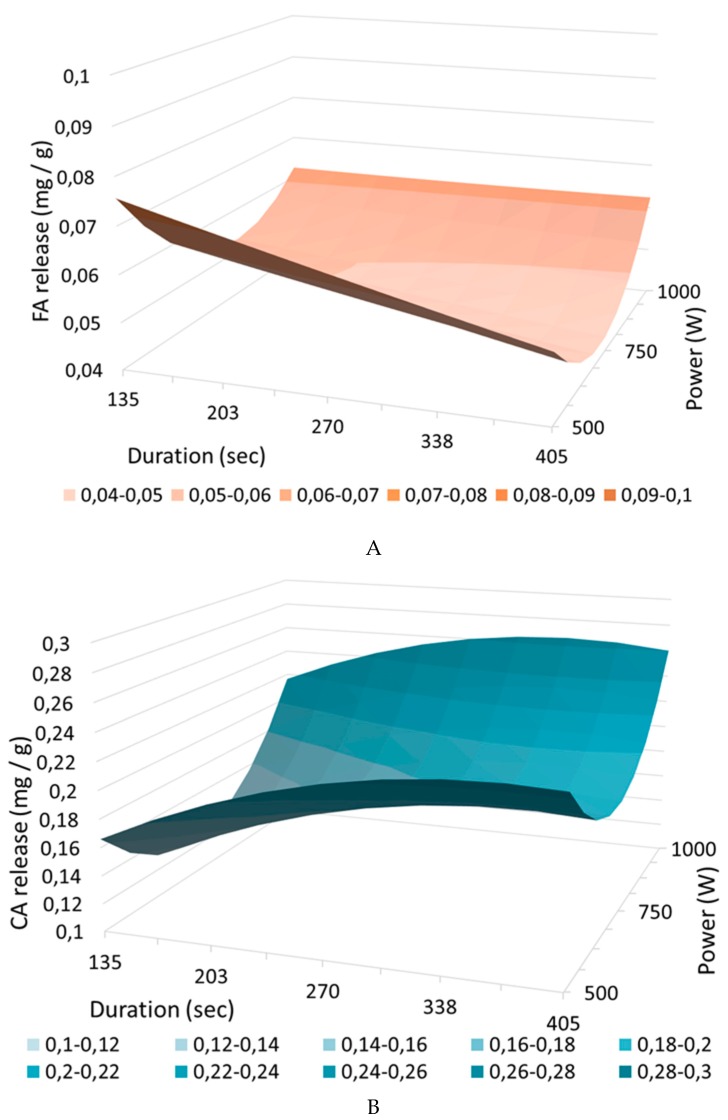
Response surface plotplan for FA (**A**) and CA (**B**) release.

Using the conditions described in 3.9, values for the center points were highly repeatable with a standard deviation of 0.3% and 0.2% for CA and FA, respectively. This provided an idea of the experimental error over the entire design of the experiments. Each *p*-hydroxycinnamic acid analysis was performed in duplicate, and the results are shown in Figure 3 and Table 4. The polynomial equations for FA (Equation (1)) and CA (Equation (2)) are the following:

(1) $$Y_{FA}=9,26-1.17X_{1}-0.36X_{2}+1.17X_{1}X_{2}+0.03X_{1}^{2}+1.89X_{2}^{2}\;R^{2}\;=\;0.749;\;F-\mathrm{value}\;=\;58.521$$

(2) $$Y_{CA}=70.50+12.37X_{1}+11.52X_{2}-2.20X_{1}X_{2}-5.81X_{1}^{2}+16.09X_{2}^{2}\;R^{2}\;=\;0.995;\;F-\mathrm{value}\;=\;0.003$$

As indicated in Joglekar and May [31], if the R² of a model is superior to 0.8, the model matches the experimental data. The R² obtained in the case of CA is high, which corresponds to an excellent regression: the model explains 99.5% of the experimental data. Moreover, the F-value calculated for CA (0.003) is very much lower than the 95% confidence level Fischer parameter (18.51), thus meaning that the model is relevant in explaining CA release results. On the contrary, the small R² (0.749), and the high F-value (58.521) obtained in case of FA indicated that the model does not fit the data, and results cannot be easily exploited using the model.

**Figure 4:**
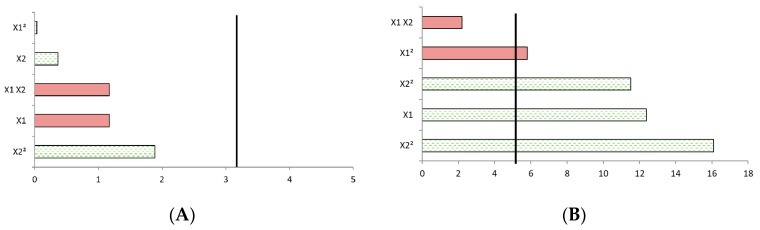
Pareto diagram for FA (**A**) and CA (**B**). X1 corresponds to duration, X2 corresponds to incident power, red surfaces indicate a negative impact, green surfaces indicate a positive impact, the vertical line corresponds to the value to be exceeded so that the response is significantly impacted by the parameter (*p* < 0.05%, corresponding to 3.18 in the design developed)

Using Pareto charts, the effects of each individual term of the polynomial equations are presented on Figure 4 for FA release (Figure 4A) and CA release (Figure 4B). A term is considered to have a significant impact on acid release if it exceeds the black vertical bar (*p* < 0.05%) corresponding to 3.18 in the study conditions. Analyzing each term of the polynomial equation permits highlighting interactions between parameters. In the case of FA (Figure 4A), none of the parameters significantly impacted FA release, which can be due to the design itself not fitting the data. Maybe FA acid release is not impacted by power nor by treatment duration: temperature could be another parameter to test, especially under pressured conditions. In that case, care must be taken not to obtain significantly negative interactions between new parameters. Another hypothesis is that the studied domain is too restrictive to obtain a significant trend on FA release, but expanding the range may not be a good option from an industrial point of view, as increasing power or processing time consumes a lot of energy and will certainly not achieve high yield. Thus, no optimum is visible on the three-dimensional (3-D) representation (Figure 3A). On the contrary, for the CA Pareto chart (Figure 4B), all coefficients have a significant impact on CA release except concerning interaction between power and duration. Another method to find the optimum of the model would have been to perform an optimization from close to close by fixing a variable (for example, the duration) and varying the other (the incident power) variables, as they have no significant effect on each other. All the other variables have a significant positive or negative impact on CA release (t-value >3.18), which supports the choice to use this model with these variables and this domain. Incident power and duration both have a significant positive impact on CA release, and this impact is similar for both variables (both coefficients close to 0.02). Thus, by increasing the incident power and duration, it is expected that the release of CA increases, and especially with regard to the increase in incident power, as its quadratic interaction is high. These comments on CA release are also visible on the response surface 3-D plot (Figure 3B), where it is noteworthy that as the power or the duration increases, the value of released CA steps up. Finally, it would be wise to choose processing times among the high tested values and a sufficiently high power to obtain good yields while respecting a possible and viable economic process.

It would be interesting to test stronger conditions, for example 1500 W and a duration of 810 s. However, these conditions were experimentally difficult to reach above a certain power and duration; the material boiled strongly in the reactor, and liquid escaped. For example, this boiling phenomenon was observed in the case of NaOH at too-high power levels or with water with a longer treatment duration. In addition, a longer reaction time would only be advantageous from an industrial point of view if it would greatly increase *p*-hydroxycinnamic acid yields. Working under pressure to reach higher temperatures is also a possible solution, but the destruction of products is an important trend to be controlled [15]. Finally, over time, some inhibitors may form, creating problems for the eventual rest of the process in the case of bioprocesses [32].

#### 2.3.2. *p*-Hydroxycinnamic Acids Release Depending on Incident Power and Treatment Duration

According to Table 4, CA release was always higher than FA release: under the most severe conditions (1000 W and 405 s), 1.38% FA and 2.0% CA were released. This observation is valid for all operating conditions. The initial high presence of CA (13.1 mg CA/g and 4.2 mg CA/g) in raw corn stalks can explain the higher CA released yields. Moreover, FA release cannot be optimized by varying the power and duration, as these parameters have no impact according to the 3-D plots (Figure 3). This is consistent with Pinela’s analysis [33]: On tomato fruits, the incident microwave power had no effect on *p*-hydroxycinnamic acids release, and a Box–Behnken design was carried out using four independent variables (duration, temperature, ethanol concentration, and liquid–solid ratio). With a treatment time of 3 min, a temperature of 140 °C, and no ethanol, 23 mg CA/g was removed. Tomatoes are naturally richer in *p*-hydroxycinnamic acids than corn stalks, and molecules are more accessible. Carniel et al. [18] reached the same conclusion using *Physalis angulate*: incident power and time had no effect on the total phenolic compound released, but the parameters range tested were very tight: between 10 and 30 W for 40 to 60 s. Moreover, the Folin–Ciocalteu colorimetric method was used to determine the total phenolic compounds, which is a different method from the one used in the present study, and the detected molecules are not the same. The Folin–Ciocalteu colorimetric method permits detecting all phenolic compounds contrary to HPLC, which detects selectively *p*-hydroxycinnamic acids.

ANOVA was performed to determine the significant impact of time and/or power on FA and CA release (Table 5). According to Table 5, CA release was significantly impacted by both time and power: CA release could be optimized depending on these parameters. With 405 or 270 s treatment time and 1000 W, CA release significantly increased, and reached 0.260 mg/g. This result is higher than the one obtained with NaOH without microwave treatment (0.173 mg/g). Thus, CA release increased with microwave treatment, and the results obtained with Equation (2) were confirmed as CA release having increased with time exposure and microwave power. On the contrary, FA release was not significantly impacted by the different power tested nor by the treatment duration. This is consistent with the results obtained in the previous section. To conclude, it appeared that 1000 W and 405 s were good options for microwave treatment with feasible conditions.

#### 2.3.3. *p*-Hydroxycinnamic Acids Release Depending on Power Density

**Figure 5:**
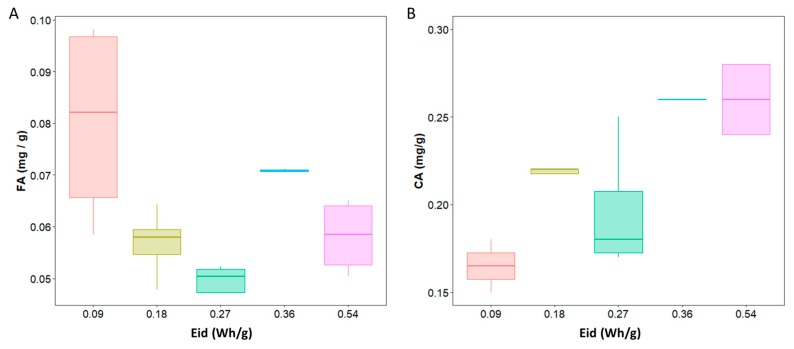
Ferulic (**A**) and coumaric (**B**) acids release depending on incident energy density.

**Figure 6:**
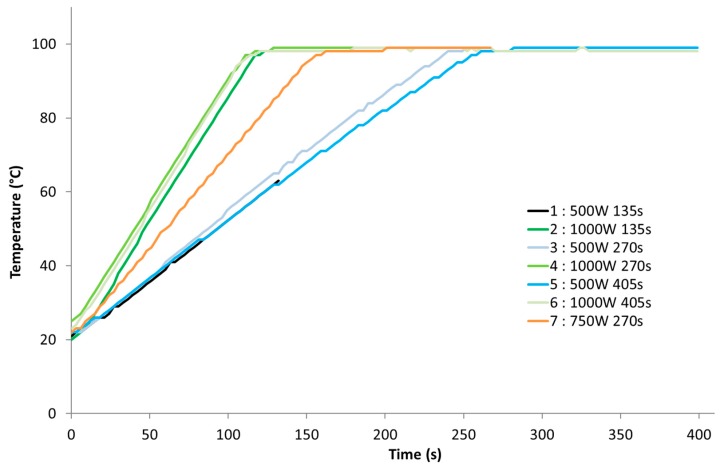
Temperature rise during response surface methodology (RSM) experiments.

An important point to elucidate was the effect of absorbed energy density (Wh/g) on *p*-hydroxycinnamic acids release: lignocellulosic biomass may react differently under various energy densities. The different energy densities tested are presented in Table 6, and a summary for each energy density is represented in Figure 5. The corresponding temperature rise graphs are shown in Figure 6. The maximum temperature of 100 °C is reached twice as fast at 1000 W as at 500 W. It is important to note that with a treatment time of 135 s and under 500 W, the reaction medium does not reach 100 °C but rather only 63 °C, which may explain the ineffectiveness of this treatment in terms of phenolic acid release. On the contrary, during 1000-W treatments, the temperature of 100 °C is reached earlier, and the material remains at 100 °C for a longer period compared to 500 W.

From Figure 5, concerning CA, the higher the energy density, the higher the *p*-hydroxycinnamic acid release (*p* = 3.44 × 10^−5^). Nevertheless, in the case of FA, it was not as simple, and no trend could be noticed (*p* = 0.274). These results are complicated to confirm, because to our knowledge, no other literature article has studied the energy density applied to the product. As a partial conclusion, to release more coumaric acid, it would be necessary to increase the energy density applied to the material either by increasing the incident power or by varying the amount of material introduced into the reactor. In all the cases tested, whatever the time, the power, or energy density, yields obtained were low (<2%). A different hypothesis could explain these low yields, especially in the case of FA.

A hypothesis is that the free acids liberated during the treatment were degraded between 80 °C and 100 °C. Tests with known ferulic and coumaric acids concentrations diluted in ethanol and submitted to the following operational conditions were performed: 500 W and 1000 W for 540 s (results not shown). A temperature of 80 °C was reached after 200 s of treatment. FA and CA concentrations detected after HPLC were similar to the ones injected: *p*-hydroxycinnamic acids were not destroyed or evaporated during treatment. This was consistent with the study of Liazid et al. [34] demonstrating that ferulic acid degradation did not occur at temperatures lower than130 °C.

Another hypothesis takes into account the chemical bonds between acids and walls. FA can be present in a variety of forms in the cell [35]. FA may be present in free form or covalently linked to lignin by ether links and to hemicelluloses by ester links forming a bridge between those two parietal polymers. FA could also be only linked to hemicelluloses by ester links (Figure 1). We suppose that ether links are more difficult to access because they are “hidden” in lignin polymers. Moreover, ether links are also difficult to break, as they are not hydrolyzed after a night with NaOH 2N. The FA release measurement was carried out on the liquid after treatment without any additional alkaline treatment: the small amounts could represent only free FA or even simple esterified FA, because etherified FA was protected by parietal components [17]. In addition, the treatment conditions implemented in this study are very soft (short duration and moderate temperature) to allow these bonds to be broken: only a few minutes in water compared to a full night in sodium hydroxide. On the contrary, CA is only linked to parietal components (both hemicellulose and lignin) by esters links: these covalent links are more easily accessible, as they are not trapped in the complex structure of the wall, and they are easier to hydrolyze and release [35]. Finally, CA are more abundant in raw corn stalks than FA. Since CA are more extracted (in mg/g) than FA and the extracting yields are similar among FA and CA, an effect of abundance in the release of CA could be hypothesized. By analyzing the composition of the walls, a modification could be detected in comparison with the raw material that would explain these low yields.

#### 2.3.4. Effect of Operational Conditions on the Pretreated Biomass Composition

**Figure 7:**
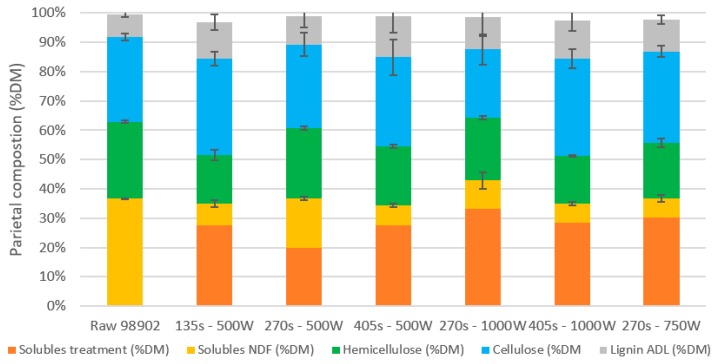
Pretreated biomass composition after RSM plan (mean of duplicate analysis).

The pretreated biomass was analyzed with the Van Soest method (Figure 7). Figure 7 represents the biomass composition after the RSM plan. It appears that biomass composition was not modified by the different operational conditions tested (*p* > 5%) when compared to the composition of the raw material. Similar results in the Klason lignin method (results not shown) confirmed that the polymer composition was not modified during microwave processing. Despite the harsher microwave conditions compared to the previous part, the composition of the biomass is still not affected. This means that the same conclusions as previously can be assumed: the conditions tested are still not effective in breaking ester and ether links and releasing phenolic acids in the liquid phase. A more precise analysis of the links between lignin units by thioacydolysis would make it possible to highlight a change in the structural organization of the plant. It must be notified that there was always an average of two grams of solubilized material during processing, corresponding to a large part of the material solubilized during the first stage of the Van Soest method on raw corn stalks.

### 2.4. Microwave Optimum Conditions Applied to Miscanthus

Based on the results from the previous paragraphs, the microwave conditions that released the most coumaric acid were chosen, as no optimization was performed for ferulic acid release. The best conditions (1000 W for 405 s) were applied to another biomass in order to find out if similar yields could be achieved (Table 7). Miscanthus was chosen because in France, it benefits the energy crop subvention in order to implement “land under industrial set-aside”. Miscanthus, similar to corn stalk, is a poaceae biomass that is very different both in terms of cell wall content (%NDF) and wall composition. Miscanthus GIB was submitted to 1000 W for 405 s with the same operational conditions as those described in Section 2.3. Control and conventional heating were also performed in duplicate on miscanthus.

From Table 7, for miscanthus stalks, 0.58% FA and 3.89% CA were recovered in comparison with corn stalks with 1.38% FA and 1.97% CA. The FA yield from miscanthus is much lower than the one from corn: more than 50%. Raw miscanthus is poorer in FA than raw corn (Table 1), which can explain the low amount of FA released during the treatment. However, we have previously demonstrated that microwave treatment, under the tested conditions, has only a limited impact on ferulic acid release, which limits the interpretation of this result.

On the contrary, CA yield in miscanthus is twice as high as in corn, in spite of the very low initial content of CA in raw matter (0.65 mg CA/g miscanthus). As explained earlier, these differences may be explained by the chemical bonds impacted, and miscanthus CA must certainly be present in free form in cells. Moreover, by comparing miscanthus microwave extraction yields with miscanthus conventional or control extraction yields, it appeared that the microwave treatment promoted the CA extraction, but the FA release remained unchanged, which was in agreement with the previous parts: CA being significantly released following the microwave treatment compared to FA.

In the tested conditions, only 0.8 g out of the 10 g of miscanthus were solubilized during the treatment, contrary to corn (2 g), which can be explained by the high initial NDF percentage in miscanthus stalks compared to NDF in corn stalks (95% DM and 65% DM, respectively). The final miscanthus composition was the following, according to the Van Soest method: 4.3% (±0.1%) soluble content, 18% (±3%) hemicellulose, 53% (±3%) cellulose, and 15.9% (±0.8%) ADL, which was not significantly different from the initial composition. These results differed from Boonmanumsin et al. [36], who experimented with microwaves combined with NH_4_OH (1% *w/v*) on miscanthus for 15 min and 300 W. *p*-hydroxycinnamic acids were not analyzed; instead, they examined monomeric sugars. In optimum conditions, up to 25.6 g sugars/100g biomass were released (principally under xylose form). The extracting solvent, the final temperature, and the duration were the main differences between the two experiments. It is always difficult to compare results from different microwave studies as various parameters may differ at the same time (duration, solvent, temperature, pressure, ratio, substrates) and may affect microwave pretreatment efficiency. As a matter of fact, general statements cannot be made for all plant materials because of their diversity in structure and composition. Processing conditions must be defined and optimized taking into account the lignocellulosic matrix and the sought-after molecules or applications [33]. Strictly speaking about *p*-hydroxycinnamic acids release, most of the studies are focusing on plant stalks, but rhizome cut in small pieces has also been investigated in the case of Scirpus holoschoenus [37]. When 20 mL of acetone 56% was added to 1 g rhizome and put under 600 W for 70 sec, up to 30 mg of gallic acid equivalent/g were extracted. Similarly, Galan et al. [38] extracted 162 mg gallic acid equivalent/g from sea buckthorn leaves after 450 s of treatment. Finally, 62 µg FA/g was extracted from Soybeen seeds after 75-W treatment for 10 min. These results highlight the importance of the raw material used during the treatments and the variability of the results obtained. The results of microwave processing are dependent on the matrix.

## 3. Materials and Methods

### 3.1. Raw Biomass

Corn stalks named F 98902 were supplied by INRA IJPB (Versailles-Grignon unit, Versailles Cedex, 78026, France) and were harvested in September 2016.

Miscanthus stalks (*M. x giganteus Britannique*, noted GIB) were supplied by INRA AgroImpact (Estrées Mons esperimental unit, Péronne, 80203, France, 49_53 N, 3_00 E) [39] and were harvested in February 2017.

Air-dried samples were coarsely crushed (Viking crusher model GE 220, STIHL, Stuttgart, Germany) and sent to the laboratory in Narbonne. Then, samples were finely ground to 1 mm using a Fritsch Pulverisette 19 grinder and sieved to retain only particles between 200 and 1000 µm. Ground and sieved samples, called “raw substrates” in the following, were kept in closed boxes in ambient air before used.

### 3.2. Chemicals

All chemicals were purchased from Merck. High-purity water (Merck Millipore Quantum TEX) was used for all pretreatments and analyses.

### 3.3. Microwave Pretreatment (MW)

Microwave pretreatments were performed with a Minilabotron 2000 microwave pilot (SAIREM, FRANCE), operating at atmospheric pressure, 2.45 GHz with a maximum power of 2 kW. All pretreatments were carried out at constant incident power level in open vessel.

According to the preliminary results (not shown), a total mass of 210 g was determined to be optimal for waves absorption based on microwave pilot configuration. Ten grams of prepared raw material with known dry matter content were transferred to a 500-mL glass reactor. Then, 200 g of liquid was added. The tested liquids (solvents) were: water, acidic water (pH = 4 obtained by adding 0.75 mmol H_2_SO_4_ to 200 mL water), alkaline water (pH = 8.5 obtained by adding 6.7 mmol NaOH to 200 mL water), or an ethanol/water mixture (50% *w/w*). This solid:liquid ratio (1:21 *w:w*) allowed correct magnetic stirring during treatments. Indeed, adequate stirring is essential for microwave treatment [15]. Samples underwent 1 h of pre-soaking at ambient temperature before microwave treatment. pH was measured at the end of this hour of contact.

Then, the reactor was closed using a glass cover connected to a refrigerant limiting solvent evaporation. A fiber-optic temperature sensor was used to monitor the temperature of the reaction medium: the maximum temperature could not exceed 100 °C in the case of aqueous solvents and 80 °C in the case of ethanol treatment because of the boiling points of these solvents. All treatments were performed at constant power and at atmospheric pressure (open vessel), in order to avoid excess temperature increase and the thermal degradation of molecules (in particular *p*-hydroxycinnamic acids) [39].

Pretreatments were performed in duplicate (excepted for the center point in the RSM plan performed in triplicate). After treatment, the reactor was air-cooled to room temperature for 15 min before weighing and pH measurement. Then, the reaction mixture was filtered through a 200-µm sieve. The solid was washed with 250 mL of deionized water to remove chemicals and by-products inhibiting enzymatic hydrolysis. The solid fraction was placed at 40 °C for seven days to dry. Then, dry matter content was measured to determine the amount of solubilized matter during processing and the solid recovery yield (% of g pretreated biomass/g raw matter). The supernatant was filtered through a cellulose filter (2.7 µm) and stored at −20 °C until further analysis.

### 3.4. Energy Calculations

Incident power varied between 500 W, 750 W, and 1000 W for 135 s, 270 s, and 405 s (Table 6). Temperature, incident power, and reflected power were recorded every three seconds during the treatment. Absorbed power was obtained using Equation (3). The total absorbed power was the sum of all the absorbed power recorded every three seconds; see Equation (4). Absorbed energy was calculated from Equation (5) by multiplying the total Pa by 3, since it was only recorded every 3 s.

Absorbed Power (Pa) = Incident Power (Pi) – Reflected Power (Pr) in W(3)

Total Pa = ∑ Pa in W(4)

Absorbed Energy density (Ead) = (∑ Pa.3)/(3600.Total mass) in Wh/g(5)

### 3.5. Conventional Heating Treatment (Conv)

A 500-mL Schott bottle containing the same reaction mixture as the microwave-treated sample was immersed in a heat-stabilized oil bath at 100 °C for 360 s, which is the reaction time necessary to reach a temperature close to 100 °C. As the bottle was closed, pressure could slightly increase during treatment without exceeding 1.3 bars. This pressure, close to atmospheric pressure, was considered to have no impact during conventional treatment. The separating protocol was the same as that described before. The conventional test allowed comparing conventional and microwave heating modes. The temperature and pH were recorded during processing.

### 3.6. Control treatment (C)

A control treatment (soaked biomass sample without any heating) was also carried out. Liquid and solid phases were separated after an hour of contact using the method described before. Pretreatment effects on biomass were evaluated by *p*-hydroxycinnamic acids release and biomass structure changes.

### 3.7. Ferulic and Coumaric Acids Analysis

Initial FA and CA amounts were determined by HPLC (method described below). A mild alkaline extraction for a night using 2 mL of NaOH 2N with 20 mg of raw matter permitted releasing esterified *p*-hydroxycinnamic acids [13,26,27]. These initial amounts (in mg/g) were used to calculate the release yields (Equation (6)).

Ferulic acid (FA) and p-coumaric acid (CA) released in the liquid phase after pretreatments were quantified in duplicate. *P*-hydroxycinnamic compounds were analyzed by HPLC using a HPLC-DAD Waters system: autosampler 717, multisolvent delivery system 600, Diode Array Detector 2996. Then, *p*-hydroxycinnamic acids were detected at 320 nm, and the peak areas were calculated by Empower3 software (Waters). The mobile phases consisted of ultrapure water/formic acid–95/5 (Solvent A) and acetonitrile/ultrapure water/formic acid–80/15/5 (Solvent B). The flow rate was 1 mL/min, and the injection volume was 10 µl. Separation was performed at 30 °C on a Waters Atlantis T3 Column, 100 Å, 5 µm, 4.6 mm × 250 mm (C18) equipped with a C18–4 × 3 mm Security Guard Cartridge (Phenomenex, France).

Then, the results—which were obtained in mg/l—were transformed to g/g DM using the collected liquid volume (l) after treatment (Equations (6) and (7)):

Collected liquid = Initial V − evaporation V − swelling V in L(6)

FA released = FA (mg/L) × Collected liquid/10gDM in mg/g(7)

Swelling was previously measured (results not shown) and corresponded to 1 mL/g DM and 0.9 mL/g DM for corn stalks and miscanthus stalks, respectively. The evaporation volume was measured by taking the mass difference between the beginning and the end of the treatment. The evaporated volume did not depend on the biomass used, but instead only on the power and duration of the treatment, as well as on the solvent. On average, for water microwave treatments, the evaporated volume can be calculated with Equation (8) with R² = 0.91:

f(duration × Incident power) = 7449.4 × evaporation V + 83002(8)

For ethanol microwave treatments, the evaporation volume was twice that obtained in water under the same conditions. For control and conventional heating, evaporation was considered negligible, as it was less than 10 mL. In any case, these mass changes in the microwave reactor over time were not taken into account in the calculations of power or energy density.

FA extraction yield was calculated as the quotient of the liberated FA/CA mass released by the total FA/CA mass in the raw substrates (equation (9) for FA).

FA yield = FA released (mg/g)/FA initial (mg/g) in %(9)

### 3.8. Biomass Composition Analysis

The impact of the pretreatment on biomass structure was evaluated using the Van Soest method [40] and Klason lignin protocol [41].

The Van Soest protocol allowed evaluating the evolution in parietal polymers content after treatment. This method is based on the mass sequential partitioning of cell walls, from most extractible to less extractible, with successive extractions using different solvents (water, neutral detergent solution, acid detergent solution, and acid 72%). After the first Van Soest step, a part of neutral detergent fiber (considered as parietal residue) was used to measure the Klason lignin content using the NREL protocol. ADL (acid detergent lignin) lignin (%DM) is the lignin obtained with the Van Soest method, and corresponds to lignin insoluble in 72% H_2_SO_4_ after a first extraction step in an acid detergent solution (ADS). Klason lignin is obtained from parietal residue and corresponds to lignin insoluble in 72% and in 4% H_2_SO_4_; the result is then reported in dry matter percentage. As explained in Hatfield and Fukushima [42], ADS can solubilize cellulose and the most accessible part of lignin (about 50%), explaining why Klason lignin is much higher than ADL, reaching twice its value. Moreover, some proteins could condense and increase the Klason lignin mass, even if this explanation is improbable in the case of grass. Finally, it was determined on a maize cell wall that Klason lignin and ADL represent two different lignin measurements: therefore, it is essential to always compare values from the same analysis [43]. The lignin part solubilized in ADS fraction was found to be correlated with β-O-4 lignin bonds. Thus, according to Zhang et al. [43], comparing Klason lignin and ADL lignin values could reveal a variation in chemical bonds within lignin.

### 3.9. Statistical Analysis and Response Surface Methodology Method

All the statistical tests were performed using R software (version 3.4.0). Microwave effects on biomass composition and on *p*-hydroxycinnamic acids release were analyzed with ANOVA and considered significant when p-value < 0.05 with residuals distributed according to a normal law. Biomass composition with Van Soest or Klason lignin were repeated twice for each sample obtained from a pretreatment. In addition, *p*-hydroxycinnamic analysis was performed in duplicate from the liquid phase of each pretreatment.

RSM allows obtaining robust results with as few experiments as possible to understand the effects of each variable on the response and find optimal conditions [44]. The plan chosen was a full factorial plan, combining mathematical and statistical techniques to model a problem and optimize the response [45]. The experimental domain was determined based on the literature, the results from experimental assays, and taking into account the experimental limits of the Minilabotron microwave pilot. Since we chose to work in an open vessel and thus without pressure, temperature was not chosen as a parameter. The two chosen parameters (z = 2) were the reaction time (X1 in seconds) and the incident power (X2 in Watt). The number of experiments (N) was determined with Equation (10):

N = z² + z + C = 2² + 2 + 3 = 9(10)

where C represents the number of center points, which was repeated three times. The experimental plan is summarized in Table 8.

Experimental data can be adjusted to a second-order model, following Equation (11):

Yi = b_0_ + b_1_ X_1_ + b_2_ X_2_ + b_12_ X_1_ X_2_ + b_11_ X_1_² + b_22_ X_2_² (11)

where Yi is the experimental response that corresponds to FA and CA release in the liquid phase (mg/g), Xi represents the studied parameters, b_0_ is the average response, b_i_ represents the linear coefficients, b_12_ is the average effect of interaction factor, and b_ij_ represents the quadratic coefficients. The model was validated or not using a Fisher test for each studied response (FA and CA). For the calculation of the different coefficients, refer to Witek-Krowiak et al. [46]. The solution of the response equations was performed using a Microsoft Excel spreadsheet.

## 4. Conclusions

The present study was designed to determine the effects of microwave irradiation on grass stalks as a pretreatment for biomass deconstruction and *p*-hydroxycinnamic acids release. Different microwave experimental conditions were set up for various incident powers, durations, and solvents. Experiments have shown that microwave power and duration had a low impact on biomass parietal composition and on FA release. CA release yields remained low, but by increasing power and duration, they could progress according to RSM. It would be interesting to implement an experimental design to understand grass biomass behavior under microwaves and to find out their real effect (radiation vs heat).

## Figures and Tables

**Table 1:** Raw matter characteristics: parietal composition analyzed by Van Soest method and Klason lignin (mean of triplicate and standard error) and alkali extraction for ferulic acid (FA) and coumaric acids (CA) (mean of duplicate and standard error). ADL: acid detergent lignin, DM: dry matter.

		Corn Stalks F98902	Miscanthus Stalks GIB Genotype
**Soluble Content (%)**		35.67 ± 1	4.81 ± 1
**Parietal Composition using Van Soest Method (%DM)**	Cell wall	64.33 ± 1	95.19 ± 1
	Cellulose	28.87 ± 0.5	55.75 ± 1.6
	Hemicellulose	26.06 ± 1	21.75 ± 1.1
	ADL	8.18 ± 1.7	16.86 ± 1.6
	Ash	1.22 ± 0.3	0.83 ± 0.5
**Klason Lignin (% DM)**		15.00 ± 0.5	24.34 ±0.2
***p-* Hydroxycinnamic Acids (mg/g DM)**	Ferulic acid	4.2 ± 0.9	2.1 ± 0.5
	Coumaric acid	13.1 ± 2.6	6.5 ± 2.3

**Table 2:** Impact of solvents and treatments on FA and CA release in liquid phase after treatments (mg/g DM) with 200/10 (*w/w*) as liquid/solid ratio. Each treatment was performed in duplicate, CA and FA measurements were duplicate for each test, and the standard deviation was expressed as a percentage.

Solvent	Reactant Concentration (mg/g liquid)	Reactant Mass (mg/g DM)	Initial pH	Final pH	Microwave Heating (mg/g DM)		Final Microwave Temperature (°C)	Conventional Heating (mg/g DM)		Final Conventional Temperature (°C)	Control (mg/g DM)	
					CA	FA		CA	FA		CA	FA
**Water**	/	/	5.3	5.1	0.201 ± 0.4%	0.047 ± 0.1%	99	0.125 ± 0.1%	0.051 ± 0.2%	69	0.085 ± 0.3%	0.057 ± 0.2%
**Acid**	0.34	7.3	3.95	3.7	0.113 ± 1.2%	0.030 ± 0.3%	99	0.071 ± 0.6%	0.027 ± 0.3%	66	0.060 ± 0.2%	0.025 ± 0.4%
**Base**	0.12	27	8.5	8.3	0.247 ± 0.1%	0.056 ± 0.1%	99	0.230 ± 0.5%	0.081 ± 0.3%	71	0.173 ± 1.4%	0.093 ± 1.6%
**Ethanol**	500	/	5.5	5.5	0.298 ± 0.6%	0.037 ± 0.4%	81	0.174 ± 0.1%	0.035 ± 0.3%	75	0.159 ± 0.5%	0.038 ± 0.5%

**Table 3:** ANOVA parameters to the significant effects on FA and CA extraction depending on treatment and solvent (CA and FA measurements realized in duplicate for each test).

Factor	CA					FA				
	d.f.	SS	MS	F	p	d.f.	SS	MS	F	p
**Treatment**	2	0.0380	0.0190	39.23	2.7 × 10^−7^ *	2	0.0005	0.0002	3.086	0.0704
**Solvent**	3	0.0747	0.0249	51.31	5.07 × 10^−9^ *	3	0.0082	0.0027	34.506	1.13 × 10^−7^ *
**Residuals**	18	0.0087	0.0004			18	0.0014	0.0001		

d.f. = degrees of freedom, SS = sum of squares, MS = mean of squares, * significant at 95% confidence level.

**Table 4:** Ferulic acid (FA) and coumaric acid (CA) release yields using the experimental design. Yields are expressed in %: mg_phenolic acid release_/mg_initial phenolic acid,_ mean of duplicate.

Assay	T Final (°C)	FA (mg/g)	FA Yield (%)	CA (mg/g)	CA Yield (%)
**1**	63	0.080 ± 2%	1.90	0.166 ± 1.1%	1.3
**2**	98	0.058 ± 0.1%	1.38	0.216 ± 0.1%	1.6
**3**	99	0.056 ± 0.8%	1.33	0.219 ± 1.1%	1.7
**4**	99	0.071 ± 0.1%	1.69	0.260 ± 0.1%	2.0
**5**	98	0.059 ± 0.9%	1.40	0.229 ± 2.8%	1.7
**6**	98	0.058 ± 0.7%	1.38	0.260 ± 1.9%	2.0
**7/8/9**	98	0.048 ± 0.2%	1.14	0.176 ± 0.3%	1.3

**Table 5:** ANOVA parameters to the significant effects on FA and CA extraction depending on treatment duration (sec) and power (W). Phenolic acid analysis in duplicate for each test condition.

Factor	CA					FA				
	d.f.	SS	MS	F	p	d.f.	SS	MS	F	p
**Duration (sec)**	1	0.011	0.01	16.59	3.6 × 10^−4^ *	1	4.2 × 10^−4^	0	2.65	0.115
**Power (W)**	1	0.009	0.009	14.15	8.3 × 10^−4^ *	1	4.6 × 10^−5^	0	0.28	0.597
**Residuals**	27	0	6.9 × 10^−4^			27	0	0		

**Table 6:** Energetic aspects for duration and incident power tested.

Total Mass (g)	Pi (W)	Pid (W/g)	Duration (s)	Eid (Wh/g)
**210**	500	2.38	135	0.089
**210**	1000	4.76	135	0.179
**210**	500	2.38	270	0.179
**210**	1000	4.76	270	0.357
**210**	500	2.38	405	0.268
**210**	1000	4.76	405	0.536
**210**	750	3.57	270	0.268

**Table 7:** CA and FA yields after optimum microwave conditions for corn and miscanthus stalks, and after control and conventional treatment for miscanthus stalks. FA and CA yields are expressed in %: mg_phenolic acid release_/mg_initial phenolic acid._

		FA Recovery Yield (%)	CA Recovery Yield (%)
**MW 1000 W–405 s**	Corn stalks	1.38 ± 0.1	1.97 ± 0.0
	Miscanthus stalks	0.58 ± 0.2	3.89 ± 0.2
**Conventional heating**	Miscanthus stalks	0.53 ± 0.0	1.94 ± 0.4
**Control (no heating)**	Miscanthus stalks	0.47 ± 0.0	1.46 ± 0.0

**Table 8:** Pretreatment conditions for the experimental design and results (mean of two measures from assays 1 to 6).

Assay	Parameter X_1_: Duration (sec)		Parameter X_2_: Power (W)	
	Code	Value	Code	Value
**1**	−1	135	−1	500
**2**	−1	135	1	1000
**3**	0	270	−1	500
**4**	0	270	1	1000
**5**	1	405	−1	500
**6**	1	405	1	1000
**7/8/9**	0	270	0	750
